# Sequencing and analysis of the complete mitochondrial genome of *Coenobita brevimanus*

**DOI:** 10.1080/23802359.2019.1643801

**Published:** 2019-07-19

**Authors:** Qi Wang, Yunjie Li, Dan Tang, Jie Wang, Jiayan Xu, Xinyi Xu, Zhengfei Wang

**Affiliations:** a Jiangsu Key Laboratory for Bioresources of Saline Soils, Jiangsu Synthetic Innovation Center for Coastal Bio-agriculture, Yancheng Teachers University, Yancheng, China;; b Jiangsu Provincial Key Laboratory of Coastal Wetland Bioresources and Environmental Protection, School of Ocean and Biological Engineering, Yancheng Teachers University, Yancheng, China

**Keywords:** Anomura, *Coenobita brevimanus*, Mitogenome, Phylogenetic

## Abstract

Coenobitidae is one of the most important families in Anomura. However, systematic classification and taxonomic studies are still limited. In this study, we determined the complete mitochondrial genome (mitogenome) of *Coenobita brevimanus* and further explored the phylogenetic relationships of Anomura. In the whole mitogenome of *C. brevimanus* (16388 bp), the AT-skew is negative and the GC-skew is positive.The phylogenetic tree was constructed using MrBayes method based on the 13 PCGs, which showed that Coenobitidae is monophyletic with maximal support value. Our phylogenetic analysis can be used to provide a basis for studies of the mitochondrial evolution of Anomura.

## Introduction

*Coenobita brevimanus* (Dana, 1852), belongs to the Coenobitidae, is a large land hermit crab (Reshmi and Bijukumar 2010). It is widely distributed on coastal forests, rocks or under the woods between the caves (Hamasaki et al. 2017). The land hermit crabs (Anomura, Paguroidea, Coenobita) mainly occur in subtropical and tropical coastal regions, have been exploited as an ornamental animal and for human consumption. Coenobitidae has been studied extensively, such as an aerial sense of smell (Harzsch and Hansson 2008), biology and ecology (Drew et al. 2010), and spermatophore diversity (Tudge 1991). However, until now, the genetics background about *C. brevimanus* was rarely reported. In the present study, we determined the complete mitochondrial genome of *C. brevimanus*.

The sequenced specimen was collected in Barru (Indonesia) on December 20th, 2018 (4°25′12″S, 119°35′50″E). The voucher specimen (No. 18122003-2) was stored at the Yancheng Teachers University. Total DNA was extracted from the muscle tissues and using the Aidlab Genomic DNA Extraction Kit (Aidlab Biotech, Beijing, China). The mitogenomes of *C. brevimanus* were sequenced by next-generation sequencing (Illumina HisSeq 4000, Illumina, San Diego, CA), and clean data without sequencing adapters were *de novo* assembled by the NOVOPlasty software (Dierckxsens et al. 2017). The mitogenome of *C. brevimanus* is a closed circular molecule of 16,388 bp in size. The gene content is typical of other decapoda mitochondrial genomes, including 13 PCGs, 2 rRNA genes, 22 tRNA genes (one for each amino acid, two for Leucine and Serine), and a major non-coding region known as the CR. Most genes (19 of 37) are encoded on the heavy (+) strand, while the rest 18 genes (5 of the 13 PCGs, 11 tRNAs, and 2 rRNAs) are situated on the light (−) strand. The mitogenome of *C. brevimanus* contains 1849 bp of intergenic spacer sequence, distributed in 27 regions, ranging from 1 to 1375 bp in size. The nucleotide composition of the mitogenome of *C. brevimanus* is as follows A = 4408 (26.9%), T = 6735 (41.1%), G = 3655 (22.3%), and C = 1590 (9.7%). The AT-skew for the whole mitogenome is negative, and the GC-skew for the mitogenome is positive. The mitogenome of *C. brevimanus* has been deposited in GenBank under accession number MN030160.

**Figure 1.**
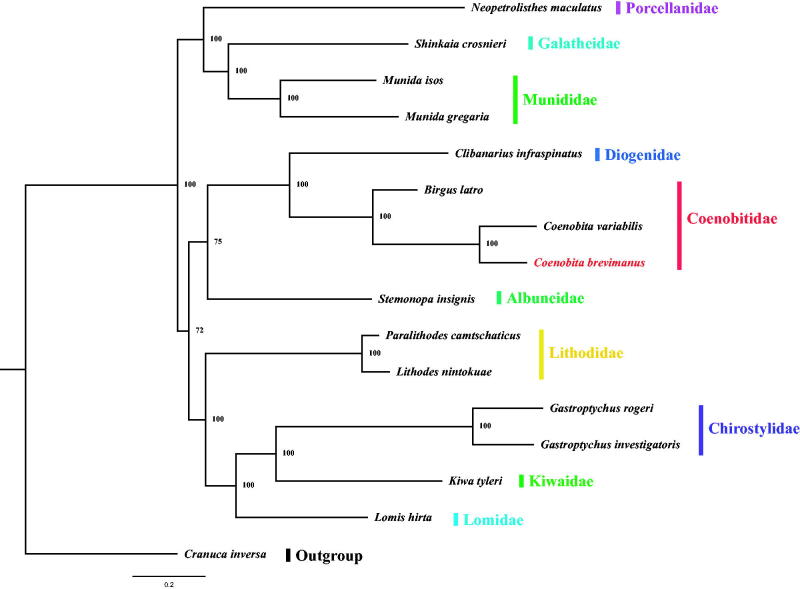
Phylogeny of Anomura based on nucleotide sequences. The phylogenetic tree was inferred from the nucleotide sequences of 13 mitogenome PCGs using BI methods. Numbers on branches indicate posterior probability (BI). *Cranuca inversa* was used as outgroup. The genbank accession numbers for all of the sequences is listed as follows: *Neopetrolisthes maculatus* NC_020024.1, *Shinkaia crosnieri* NC_011013.1, *Munida isos* NC_039112.1, *Munida gregaria* NC_030255.1, *Clibanarius infraspinatus* NC_025776.1, *Birgus latro* KY352241.1, *Coenobita variabilis* KY352236.2, *Coenobita brevimanus* MN030160, *Stemonopa insignis* KY352240.1, *Paralithodes camtschaticus* NC_020029.1, *Lithodes nintokuae* NC_024202.1, *Gastroptychus rogeri* KY352238.1, *Gastroptychus investigatoris* KY352237.1, *Kiwa tyleri* NC_034927.1, *Lomis hirta* KY352239.1, *Cranuca inversa* NC_039111.1.

The phylogenetic relationship of Anomura was constructed on the concatenated set of nucleotide sequences of the 13 PCGs from the mitochondrial genomes of 15 Anomura species and one outgroup (*Cranuca inversa*). The phylogenetic tree was performed with the Bayesian interface (BI) method using MrBayes v3.2.6 (Huelsenbeck and Ronquist 2001). The result showed that *C. brevimanus* was sister to *Coenobita variabilis* and then clustered with *Birgus latro* in Coenobitidae with maximal support value (BI posterior probabilities [PP] = 100, Figure 1). In addition, Anomura was divided into two main branches in our Phylogenetic tree, which was identified with the previous research (Lee et al. 2016). The first clade comprised 3 families (Porcellanidae, Galatheidae, and Munididae) and the second clade contained 7 families (Diogenidae, Coenobitidae, Albuneidae, Lithodidae, Chirostylidae, Kiwaidae, and Lomidae). The phylogenetic relationships of these 10 families exhibit as ((Galatheidae + Munididae) + Porcellanidae) + ((((Chirostylidae + Kiwaidae) + Lomidae) + Lithodidae) + ((Coenobitidae + Diogenidae) + Albuneidae)). Our newly acquired mitochondrial genome data and phylogenetic results can be better used to provide a basis for studies of the mitochondrial evolution of Anomura.
